# High-Throughput Molecular Modeling and Evaluation of the Anti-Inflammatory Potential of Açaí Constituents against NLRP3 Inflammasome

**DOI:** 10.3390/ijms25158112

**Published:** 2024-07-25

**Authors:** Elaine Cristina Medeiros da Rocha, João Augusto Pereira da Rocha, Renato Araújo da Costa, Andreia do Socorro Silva da Costa, Edielson dos Santos Barbosa, Luiz Patrick Cordeiro Josino, Luciane do Socorro Nunes dos Santos Brasil, Laura Fernanda Osmari Vendrame, Alencar Kolinski Machado, Solange Binotto Fagan, Davi do Socorro Barros Brasil

**Affiliations:** 1 Laboratory of Modeling and Computational Chemistry, Federal Institute of Education, Science and Technology of Pará (IFPA) Campus Bragança, Bragança 68600-000, PA, Brazil; elaine.rocha@ifpa.edu.br; 2 Laboratory of Biosolutions and Bioplastics of the Amazon, Graduate Program in Science and Environment, Institute of Exact and Natural Sciences, Federal University of Pará (UFPA), Belém 66075-110, PA, Brazil; renato.costa@ifpa.edu.br (R.A.d.C.); ac165051@gmail.com (A.d.S.S.d.C.); barbosaedielson68@gmail.com (E.d.S.B.); luciane.brasil@uepa.br (L.d.S.N.d.S.B.); davibb@ufpa.br (D.d.S.B.B.); 3 Graduate Program in Chemistry, Federal University of Pará (UFPA), Belém 66075-110, PA, Brazil; 4 Laboratório de Planejamento e Desenvolvimento de Fármacos, Instituto de Ciências Exatas e Naturais, Universidade Federal do Pará, Belém 66075-110, PA, Brazil; patrickcordeiro11@gmail.com; 5 Laboratory of Molecular Biology, Evolution and Microbiology, Federal Institute of Education, Science and Technology of Pará (IFPA) Campus Abaetetuba, Abaetetuba 68440-000, PA, Brazil; 6 Graduate Program in Nanosciences, Franciscana University, Santa Maria 97010-032, RS, Brazil; laura.o.vendrame@gmail.com (L.F.O.V.); alencarkolinski@gmail.com (A.K.M.); solange.fagan@gmail.com (S.B.F.)

**Keywords:** NLRP3 inflammasome, açaí (*Euterpe oleracea*), anti-inflammatory, molecular docking, molecular dynamics simulations, MM/GBSA, in silico toxicology

## Abstract

The search for bioactive compounds in natural products holds promise for discovering new pharmacologically active molecules. This study explores the anti-inflammatory potential of açaí (*Euterpe oleracea* Mart.) constituents against the NLRP3 inflammasome using high-throughput molecular modeling techniques. Utilizing methods such as molecular docking, molecular dynamics simulation, binding free energy calculations (MM/GBSA), and in silico toxicology, we compared açaí compounds with known NLRP3 inhibitors, MCC950 and NP3-146 (RM5). The docking studies revealed significant interactions between açaí constituents and the NLRP3 protein, while molecular dynamics simulations indicated structural stabilization. MM/GBSA calculations demonstrated favorable binding energies for catechin, apigenin, and epicatechin, although slightly lower than those of MCC950 and RM5. Importantly, in silico toxicology predicted lower toxicity for açaí compounds compared to synthetic inhibitors. These findings suggest that açaí-derived compounds are promising candidates for developing new anti-inflammatory therapies targeting the NLRP3 inflammasome, combining efficacy with a superior safety profile. Future research should include in vitro and in vivo validation to confirm the therapeutic potential and safety of these natural products. This study underscores the value of computational approaches in accelerating natural product-based drug discovery and highlights the pharmacological promise of Amazonian biodiversity.

## 1. Introduction

The search for bioactive compounds in natural products represents a promising field for discovering new molecules with pharmacological activities. The chemical richness of these products, highlighted by their diverse biological activities, emphasizes the importance of efficiently and systematically exploring these natural resources. However, identifying and developing natural products with pharmacological activity requires innovative approaches and predictive strategies [1].

Faced with the possibility of future pandemics and diseases, drug discovery should capitalize on the available natural health products to find innovative medicines. These compounds are a significant source of active compounds, providing many molecules approved for clinical use or serving as starting points in the synthesis and development of biologically active molecules. It is noteworthy that nature has selected and optimized chemical structures for compounds enriched with biological functions over millions of years. However, identifying and developing pharmacologically active natural products is a challenge. To overcome these challenges, a wide range of computational approaches have evolved in recent years, and the number of applications of these approaches to enhance and expedite the discovery of natural product-based drugs is increasing [2,3,4,5,6].

Molecular modeling has been used in the pharmaceutical industry for years to predict how a new molecule might function based on research conducted with structurally similar molecules. This approach can help reduce costs and determine which new molecules to test and target for commercialization [7,8,9,10,11].

The vast and unique biodiversity of the Amazon Rainforest constitutes a significant source of natural resources, rich in pharmacologically active plants. These plants can serve as a basis for the research and development of new medicines. In rural and riverside communities in the region, various plants have traditionally been employed in the prevention and treatment of a wide range of symptoms and diseases [12,13,14,15,16,17,18]. The tropical forests of the Amazon are recognized as home to the greatest plant diversity globally, with approximately 30 thousand plant species. Despite many Amazonian plants being investigated for secondary metabolites, it can be stated that our current understanding of the chemical diversity of this plant biome encompasses only a minuscule fraction of this wealth [19,20,21].

The native populations of this region traditionally consume a variety of native fruits, some with worldwide notoriety, such as açaí (*Euterpe oleraceae*, Mart.), which, in addition to its energy value, presents a variety of biological properties [22]. Açaí is a palm tree, belonging to the *Arecaceae* family, that grows abundantly in north Brazil, mainly in the regions of Pará, Amazonas, Tocantins, Maranhão, and Amapá [23]. The fruits of this vegetation are rich in sugars, fatty acids, vitamins, minerals, flavonoids, and polyphenols, being widely used by the food industry in the production of beverages, sweets, and ice creams [23,24,25].

From this fruit, açaí oil can be extracted, composed mostly of saponifiable material: fatty acids, and long and unsaturated chains with oleic acid as the major constituent [26]. In lower concentrations, there are unsaponifiable compounds such as phytosterols (beta-sitosterol, stigmasterol, and campesterol), used as adjuvants in cosmetics for preventing skin aging, and phenolic acids, possessing antioxidant properties, implying extensive cosmetic and pharmacological applications for this fruit [27,28,29,30]. Studies affirm that açaí has antidiarrheal [31], anti-inflammatory and antinociceptive effects [32], inhibitory effect against *Staphylococcus aureus* [33], antiproliferative action [30], antioxidant properties [34], and anti-teratogenic effect [24]. It is also described in the literature for its absence of genotoxicity [35].

**Figure 1:**
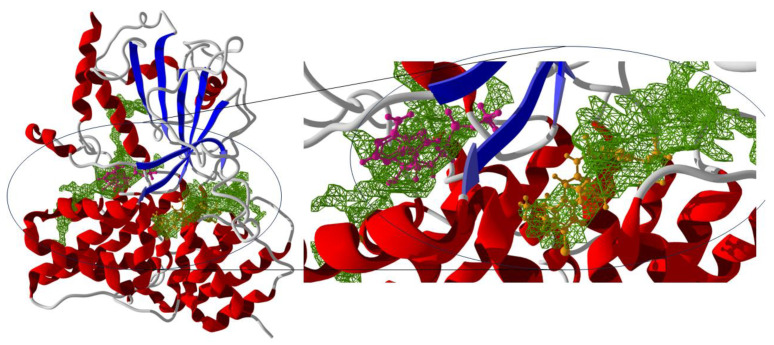
Three-dimensional structure of the NLRP3 monomer, highlighting the binding sites. Allosteric Binding Site (inhibitor RM5 in orange). ADP/ATP Binding Site (ADP in purple). Binding Pockets in green (potential binding areas in the protein structure at their respective sites). Red: Alpha helices, Blue: Beta sheets, and Gray: Loops and unstructured regions of the NLRP3 protein.

Within reports on the anti-inflammatory potential of açaí, Machado and collaborators suggested this effect in macrophage-induced inflammation with phytohemagglutinin [36]. They demonstrated that such an effect is attributed to the modulation of the NLRP3 inflammasome. The innate immune system harbors a variety of signaling receptors that discern invasive molecules and mutated self-molecules, whether excessive or present in unconventional locations. The inflammatory response represents a defensive reaction of the immune system triggered by harmful stimuli such as pathogens, allergens, or deceased cells. Imbalances in inflammation can result in persistent or systemic inflammatory disorders, with excessive inflammation associated with these challenges, while inadequate inflammation may contribute to the prolongation of pathogenic infections [37,38,39,40,41]. The inflammasome activates in response to various danger signals, whether pathogenic or sterile, playing a fundamental and innate role in the immune system. Furthermore, it has been associated as a contributor to a variety of acute and chronic diseases. The protein NLRP3 and its complexes are related to various inflammatory conditions, including neurodegenerative, autoimmune, and metabolic diseases. Recent studies indicate that NLRP3 has multiple binding pockets, which enhances its ability to recognize and bind to different ligands, thereby potentiating its role in the inflammatory response. These binding pockets include the NACHT domain, which is crucial for ATP binding and hydrolysis, and other allosteric regions that can induce NLRP3 inhibition through allosteric ligands (see Figure 1) [42,43].

The study of the NLRP3 inflammasome and its structure is a promising strategy to alleviate symptoms associated with pathological neuroinflammation and various other inflammatory conditions. However, even though the effect of the extract on NLRP3 modulation has been demonstrated, it is still unclear whether this effect is due to the synergistic action of the molecules in the extract or if it is related to a specific molecule. This uncertainty represents an innovative and crucial aspect of the study, as the precise identification of the mechanisms involved could open new avenues for research and potentially lead to the development of more effective and targeted therapies for inflammatory conditions. [36,44,45,46,47,48,49,50,51].

In this study, the chemical constituents of the hydroalcoholic extract of açaí, previously described by Machado and collaborators, will be thoroughly analyzed regarding their anti-inflammatory potential and affinity towards the NLRP3 complex. This evaluation will be conducted comparatively with two recognized experimental inhibitors, MCC950 and NP3-146 (RM5), using advanced techniques such as molecular docking, molecular dynamics simulations, free energy calculations (MM/GBSA), and in silico toxicology.

## 2. Results and Discussion

It is already very well described in the literature that toll-like receptors [52] and NLR family receptors [53,54] are directly related to the inflammatory activation process. The NLR family includes other inflammasomes despite the NLRP3, such as the NLRP1 [55] the NLRC4 [56], and the NLRP12 [57], for example. However, nowadays the most explored one is still the NLRP3 inflammasome. Many studies are showing the relationship between the NLRP3 inflammasome and several acute or chronic inflammatory diseases [58,59]. In addition, there are many in vitro investigations showing the possibility of modulating the NLRP3 artificially, which allows the development of additional studies looking forward to new anti-inflammatory agents [36,41,60]. Considering the açaí extract, there is solid scientific information that this natural health product could act as an anti-inflammatory via the NLRP3 inflammasome modulation. Machado et al. [44] were the first researchers to investigate the potential anti-inflammatory effect of açaí extract through the NLRP3 modulation. Later, Cadoná et al. [36] showed the potential of açaí extract in decreasing inflammation in microglia cells via partial inhibition of the NLRP3 inflammasome. In both studies, the authors suggest that these effects would occur due to the açaí’s chemical matrix, and this is the aspect that stimulated the conduction of our investigation through computation analysis. Unfortunately, there is still a lack of scientific information about using açaí as a modulator of other NLR family proteins instead of the NLRP3. Here we found substantial data proving that açaí chemical matrix can interact with the NLRP3, suggesting that this would be the reason how açaí acts as an anti-inflammatory.

### 2.1. Redocking

**Figure 2:**
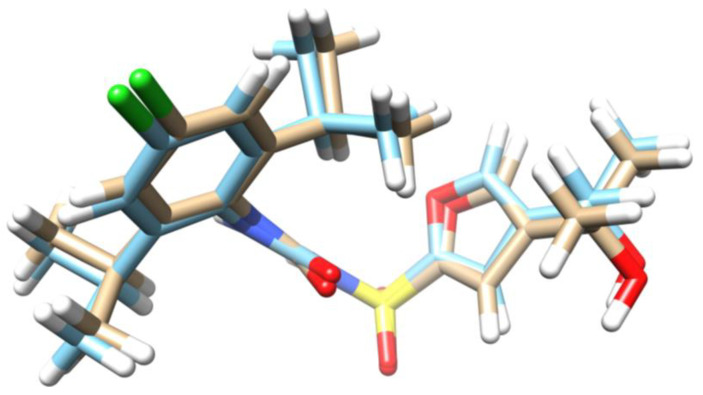
RM5 and NLRP3 Docking by MVD 5.5. This figure illustrates the docking of RM5 to the NLRP3 protein using Molegro Virtual Docker (MVD) 5.5. The blue color represents the crystal structure of NLRP3, while the light brown indicates the docking structure of RM5. The superimposition of these structures demonstrates the alignment and binding conformation of RM5 within the NLRP3 active site, validating the accuracy of the docking protocol.

The RMSD result, which was 0.391 Å, compares favorably with the experimentally determined structure to that generated by computational means, demonstrating it to be an acceptable value for the confirmation of docking protocols, as indicated in previous studies [61,62]. Traditionally, RMSD values below 2.0 Å are considered indicative of a robust solution, although this can vary depending on the size and flexibility of the ligand [63]. These findings demonstrate the software’s Molegro Virtual Docher 5.5 ability to accurately relocate the ligand RM5 (also called NP3-146) into the active site of NLRP3. Figure 2 illustrates both the experimental conformation of the aforementioned ligand and that achieved through the redocking process.

**Figure 3:**
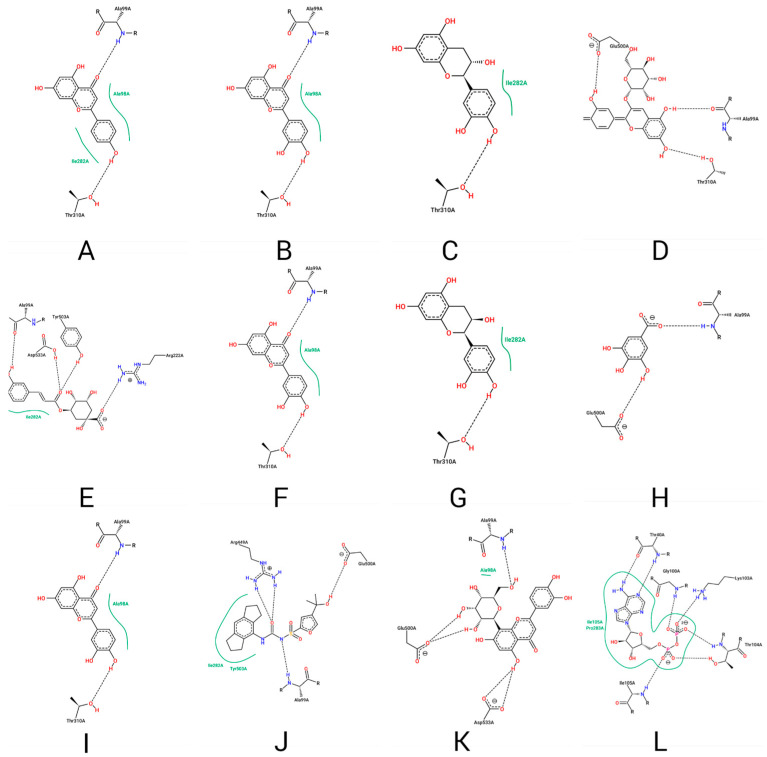
This figure shows the docking interactions of NLRP3 with several natural compounds and known inhibitors. Each subfigure (**A**–**L**) represents different ligands: (**A**)—Apigenin, (**B**)—Caffeic Acid, (**C**)—Catechin, (**D**)—Cyanidin-3-O-glucoside, (**E**)—Chlorogenic Acid, (**F**)—p-Coumaric Acid, (**G**)—Epicatechin, (**H**)—Gallic Acid, (**I**)—Luteolin, (**J**)—MCC950, (**K**)—Orientin, and (**L**)—RM5. The figure highlights key amino acid residues involved in binding, showing both shared and unique interactions among the ligands, which are crucial for understanding their binding affinities and potential inhibitory effects on NLRP3.

The compounds were evaluated using the MolDock Score scoring function to assess the affinity and interactions of these structures at the allosteric site of the NLRP3 protein complex. These were compared with those of the crystallographic ligand RM5 interactions. The results of the docking of the ligands with NLRP3 are shown in Figure 3.

### 2.2. Docking

The already known NLRP3 inhibitors share interactions with the residues: a-RM5: Thr40, Gly100, Lys103, Thr104, Ile105, and Pro283; and b-MCC950: Ala99, Ile282, Arg449, Glu500, and Tyr503. Meanwhile, in the natural compounds from the hydroalcoholic extract of açaí, the residues that share common interactions with MCC950 are Ala99, which interacts with apigenin, Cyanidin-3-O-glucoside, chlorogenic acid, *p*-coumaric acid, gallic acid, luteolin, and orientin. The residue Ile282 interacts with apigenin, catechin, chlorogenic acid, and epicatechin, and Glu500 interacts with Cyanidin-3-O-glucoside, gallic acid, and orientin.

Although initial docking analysis reveals several significant interactions in many of the natural compounds derived from açaí and the known inhibitors of NLRP3, some interactions such as Ala98, Arg222, Ile282, Thr310, and Asp535 are not exploited by MCC950 or its analog RM5. This fact indicates that, due to their distinct chemical structure, the natural compounds are likely targeting other amino acid residues to maintain their position in the allosteric site, thus corroborating other theoretical studies involving the docking of potential NLRP3 inhibitors [45,46,49,64,65,66].

Particularly, the study by McMahon demonstrates the specificity and selectivity of natural compounds in interacting with the allosteric site of NLRP3. McMahon and colleagues have shown that while MCC950 and RM5 predominantly bind to certain key residues, natural compounds exhibit affinity for other residues not commonly targeted by synthetic inhibitors. This distinction could pave the way for the development of new inhibitors that leverage these interactions, enhancing therapeutic effects and minimizing potential side effects. Furthermore, concerns about hepatic toxicity have prevented any clinical application of MCC950, underscoring the importance of exploring natural alternatives with potentially superior safety profiles [67].

The bioactive compounds extracted from the hydroalcoholic extract of açaí offer a promising route for drug development, potentially one associated with lower risks of significant side effects and reduced development costs compared to synthetic agents. The maintenance and behavior of these interactions were evaluated through molecular dynamics simulations.

### 2.3. Molecular Dynamics Simulations

Molecular dynamics (MD) simulations are widely used to investigate events in biomolecules, such as proteins. This approach is particularly useful for analyzing atomic-scale movements, which often present significant obstacles to observation by experimental methods [68]. To demonstrate the stability and conformational changes induced by the presence of ligands (10 compounds present in the hydroalcoholic extract of açaí, MCC950, and RM5), MD simulations were performed for 100 nanoseconds. Additionally, a molecular dynamics simulation of the protein in its APO form was conducted. In the present context, the “APO” form refers to a protein in its unbound state, with no ligand attached. This concept is fundamental for structural studies of proteins, as it allows for comparisons between the free form of the protein (APO) and its ligand-bound form (holo). Such comparisons are crucial for understanding how ligand binding affects the structure and function of the protein. Analyzing the APO form can provide insights into the native conformation of the protein and its intrinsic dynamics without the influence of external interactions.

**Figure 4:**
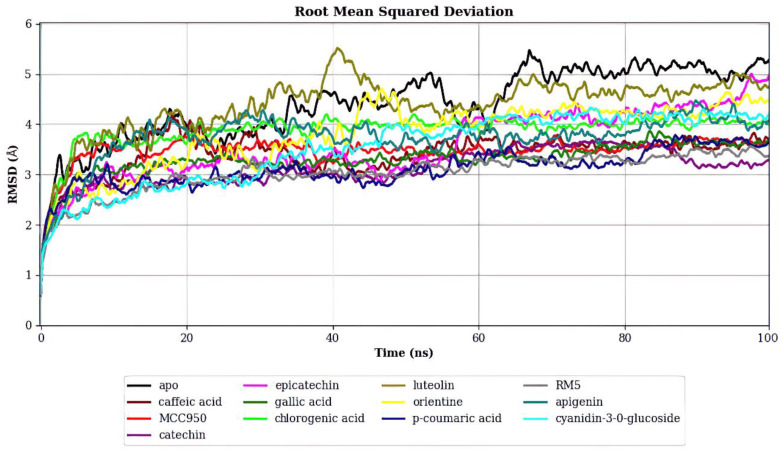
RMSD of NLRP3 in Complex with Various Ligands. This figure presents the Root Mean Square Deviation (RMSD) values over time for NLRP3 in its apo form and when bound to different ligands. Lower RMSD values indicate greater stability of the protein-ligand complex. The comparison reveals that the presence of ligands like Catechin, p-Coumaric Acid, Caffeic Acid, and Gallic Acid stabilizes the NLRP3 structure similarly to known inhibitors RM5 and MCC950, while Luteolin induces greater structural fluctuations.

As illustrated in Figure 4, the backbone RMSDs for the complexed systems converged to a lower RMSD value than the APO form, suggesting that the presence of these compounds stabilizes the protein structure. Among the açaí compounds, Luteolin exhibited the highest RMSD, yet it still converged to a lower RMSD than the APO form by the end of the simulation. Notably, Catechin, *p*-Coumaric Acid, Caffeic Acid, and Gallic Acid converged to RMSD values similar to those of the inhibitors RM5 and MCC950 (also see Table 1).

**Figure 5:**
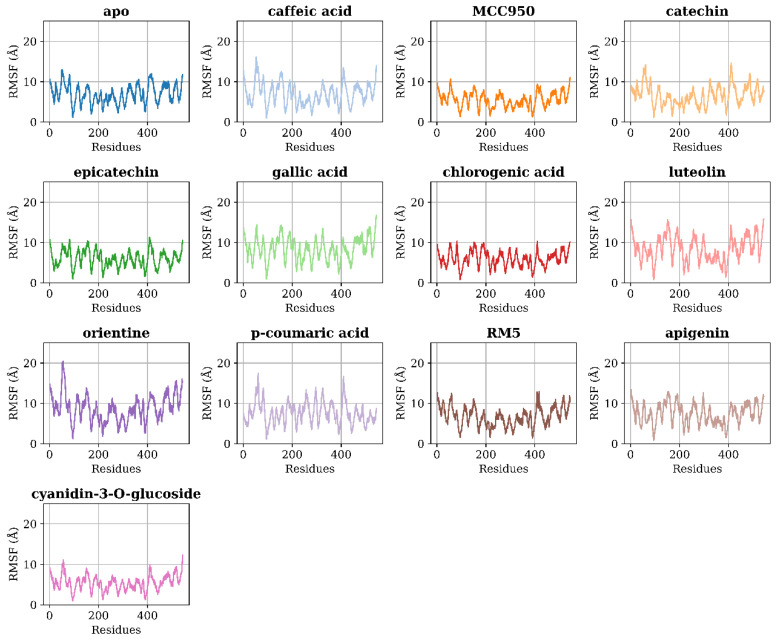
RMSF of NLRP3 Residues in Complex with Various Ligands. The Root Mean Square Fluctuation (RMSF) values plotted for each ligand demonstrate the flexibility and dynamic behavior of individual NLRP3 residues. Lower fluctuations indicate regions that interact with ligands and help stabilize them. Notably, residues like Arg43 and the region between Val94 and Ile101 exhibit low fluctuations, suggesting strong interactions with the ligands, crucial for their inhibitory potential.

Local variations within a protein can be quantified through the Root Mean Square Fluctuation (RMSF), a crucial metric used to detail specific changes along the protein chain. This measure is particularly effective for examining the flexibility and dynamic behavior of individual residues within the protein structure [69]. Figure 5 shows the RMSF values plotted separately for each ligand.

Overall, lower fluctuations can be observed in residue Arg43 and the region comprised between residues Val94 and Ile101. This region is protected by a loop and is located near the NLRP3 binding site. These fluctuations likely indicate that this region interacts with the proposed ligands and helps stabilize them. Another region with low fluctuation is the one comprised of residues Arg123 and His131, but this region is far from the binding site, and its low fluctuation is due to its deep location within the protein, protected by other residues.

In Table 1, the behavior of the solvent-accessible surface area (SASA) is shown, which is used to determine the hydrophobicity of the protein in contact with the solvent. SASA assessment is crucial not only for measuring the interaction between the protein and the solvent but also for providing insights into the energetic properties of biological macromolecules, influencing the understanding of their stability and behavior in solution [70,71]. In molecular dynamics simulation, the SASA calculation helps to identify which parts of the molecule are exposed to the solvent and which are protected inside the structure. This can be useful for studying processes such as the formation of protein-ligand complexes, the stability of proteins, and the interaction of proteins with other molecules. [72,73].

The results show that the average SASA for the protein in its APO form is higher than for the forms bound with different ligands. Specifically, the APO form has an average SASA of 27,634.13 Å^2^, while the systems with Apigenin, Caffeic Acid, Catechin, and Chlorogenic Acid have average SASA values of 26,069.83 Å^2^, 26,224.30 Å^2^, 25,698.55 Å^2^, and 26,859.50 Å^2^, respectively. This reduction in SASA in the bound systems indicates that the ligands are occupying regions of the protein surface that were previously exposed to the solvent, resulting in a smaller solvent-accessible surface area.

Furthermore, the standard deviation values of SASA are comparable across the different systems, suggesting that the variability in solvent accessibility is not significantly affected by ligand binding. These findings suggest that interaction with the ligands causes a conformational stabilization of the protein, reducing its exposure to the solvent and possibly conferring greater structural stability to the protein-ligand complex. Additionally, regions of the protein that showed low RMSF and low SASA in the bound form indicate that these areas are deeply located within the protein and protected by other residues, contributing to the structural stability of the protein-ligand complex as mentioned earlier.

Another pertinent analysis is the Radius of Gyration (Rg), a measure that describes the spatial distribution of the parts of a molecule around its center of mass. It is often used to assess the compactness of a protein or other macromolecule during simulation. It is crucial for studying protein folding, the process by which proteins assume their functional three-dimensional shape, and helps understand the aggregation of molecules, such as in the formation of micelles or protein complexes. Changes in Rg can indicate the formation or disintegration of supramolecular structures [74,75].

Rg is also useful for analyzing conformational changes in proteins in response to ligand binding, mutations, or pH changes. An increase in Rg can indicate destabilization of the protein structure, while a decrease can suggest compaction or folding [76,77]. In Table 1, it is shown that for the systems simulated with MCC950 (23.94 Å), Catechin (23.96 Å), Epicatechin (23.98 Å), and RM5 (23.97 Å), there is a decrease in the radius of gyration compared to the APO form, suggesting that the binding of these ligands may induce a more compact conformation of the protein, possibly stabilizing it. This compactness may be related to the stabilization of the protein’s inactive form, as described by Casali et al., where MCC950 binding induces conformations that are not conducive to inflammasome assembly [46].

In contrast, for Luteolin (24.73 Å), the increase in the radius of gyration suggests that the binding of this ligand may cause a more open conformation of the protein, possibly due to the induction of structural changes that increase the average distance of the atoms from the protein’s center of mass. Furthermore, Luteolin not only increases the average radius of gyration but also shows the highest standard deviation (0.25 Å), suggesting greater flexibility or conformational variation in the presence of this ligand.

This greater flexibility is corroborated by the RMSD and SASA data. The average RMSD for Luteolin is 4.38 Å, with a standard deviation of 0.58 Å, indicating significant structural variation compared to other ligands. These values suggest that Luteolin induces greater structural fluctuations, which aligns with the hypothesis of a more open conformation. Additionally, the average SASA for Luteolin is 26,876.41 Å^2^, with a standard deviation of 640.25 Å^2^. This increase in average SASA compared to the APO form (27,634.13 Å^2^) suggests that Luteolin binding exposes more of the protein surface to the solvent, reinforcing the idea that this ligand induces a more open and solvent-accessible conformation, another plausible hypothesis is that this molecule is undergoing a reconfiguration process at the NLRP3 site.

The unique behavior of Luteolin, which seems to activate NLRP3, opens several research directions. From elucidating the activation mechanisms to developing new allosteric modulators. Conducting future accelerated molecular dynamics studies, in vitro assays, and in vivo evaluations will be crucial for significantly advancing the understanding of inflammasome regulation and its therapeutic potential.

Casali’s analysis showed that the presence of MCC950 alters the coordination between protein domains. In the present data, ligands that reduce the radius of gyration may be inducing a similar effect, decreasing overall flexibility, and favoring inactive conformations. Similarly, a reduction in SASA observed with ligands may correlate with greater structural compactness and reduced solvent accessibility, consistent with the stabilization of inactive or less accessible forms.

The RMSD, SASA, and Rg data are consistent with findings from other recent studies investigating the molecular dynamics simulations of NLRP3 inflammasome and its inhibitors, offering a comprehensive view of how different ligands influence the structure and stability of NLRP3 [45,48,78]. Structural stabilization and reduction in flexibility and solvent accessibility in the presence of ligands support the idea that these ligands promote inactive and more stable conformations of NLRP3, preventing its activation and oligomeric function. These findings are crucial for the development of effective NLRP3 inhibitors and provide a solid foundation for future research aimed at reducing and/or regulating inflammatory activity.

### 2.4. MM/PBSA Binding Free Energy

To evaluate the binding affinity and probable inhibitory activity of the components of the hydroalcoholic extract of açaí and two known inhibitors (RM5 and MCC950) against NLRP3 in the protein-ligand complexes, binding free energy calculations were performed. The calculated results of the binding free energy from MM/GBSA and MM/PBSA are presented in Table 2 below.

From the analysis of the results, it was observed that the compounds RM5 and MCC950 exhibited binding energies nearly twice as low as the average energies of the natural compounds from açaí (ΔG_bind_ = −46.5965 and −42.4209 kcal/mol, respectively), indicating a high binding affinity. Among the açaí-derived compounds, notable ones include Catechin, Apigenin, Epicatechin, and Luteolin (ΔG_bind_ = −20.1292, −19.8057, −18.7737, and −18.7331 kcal/mol, respectively).

**Figure 6:**
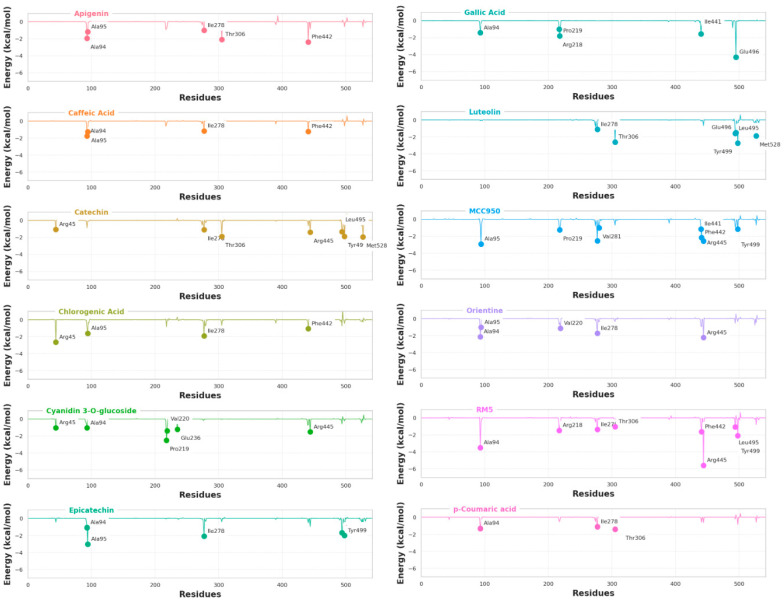
Per-residue Binding Free Energy Decomposition This figure shows the binding free energy contributions of individual residues in the NLRP3 protein for each ligand. Residues with energy contributions equal to or lower than −1.00 kcal/mol are considered significant for binding. The analysis highlights key residues such as Ile278, Thr306, Phe442, Leu495, Tyr499, and Glu496, which play a pivotal role in stabilizing the ligands within the NLRP3 binding site.

Notably, these results are consistent with the residue energy contribution analysis illustrated in Figure 6, where residues with energy contribution values equal to or lower than −1.00 kcal/mol were considered significant for the binding process. This figure presents a detailed comparison of the energy contributions of each residue in the complexes.

It is noteworthy that the known NLRP3 inhibitors form interactions with key residues in the active site of the protein complex, with RM5 interacting with Ala94, Ile278, Arg218, Thr306, Phe442, Leu495, Tyr499, and Arg445, and MCC950 interacting with Ala95, Pro219, Val281, Ile441, Phe442, Glu496, Leu495, and Arg445. This justifies their higher binding affinity. The MM-GBSA method has been effectively used in previous computational studies to estimate and select more potent NLRP3 protein inhibitors, further supporting our results [45,79,80].

Additionally, the analysis of residual interactions reveals that, while Catechin shares some interactions with the known inhibitors RM5 and MCC950, such as Ile278 and Arg445, it also explores unique residues that may contribute to its distinct binding affinity. These unique residues, such as Arg45, Thr306, Leu495, Tyr499, and Met528, can offer additional insights for the development of new NLRP3 inhibitors with unique properties.

Apigenin shares with RM5 and MCC950 the residues Ala94, Ile278, Thr306, and Phe442, while uniquely interacting with Ala95. Epicatechin shares interactions with Ala94, Ala95, and uniquely interacts with Tyr499. Meanwhile, Luteolin shares common interactions with residues Ile278, Thr306, Leu495, Glu496, Tyr499, and Phe442, and uniquely explores interaction with Met528.

By analyzing the binding interactions between the known NLRP3 inhibitors (RM5 and MCC950) and the natural compounds from açaí with the lowest MM/GBSA binding free energies (Catechin, Epicatechin, Luteolin, and Apigenin), we identified several significant interactions with key residues in the active site of the protein complex. Among these interactions, the common interactions with residues Ile278, Thr306, Phe442, Leu495, Tyr499, and Glu496 stand out, indicating that they are key residues in stabilizing molecules in the NLRP3 allosteric site.

Concerning examining the unique interactions among the natural compounds, Met528 is exclusively explored by both Catechin and Luteolin, while Tyr499 is exclusively explored by Catechin and Epicatechin. None of these unique interactions are present in all four highlighted natural compounds, but Thr306 is explored by three of them (Catechin, Apigenin, and Luteolin). This demonstrates the importance of these specific interactions in the retention and maintenance of natural compounds in the NLRP3 allosteric site.

Beyond conventional intermolecular interactions, forces such as van der Waals energy, electrostatic energy, polar solvation energy, and non-polar solvation energy also play a fundamental role in determining the total binding free energy of the complexes. As shown in Table 2, van der Waals and electrostatic energies contribute significantly to the total binding energies, while polar solvation energy exerts an unfavorable effect on the binding energy.

These data correlate with the findings discussed in the previous section. The reduction in SASA, especially in Catechin, suggests that these ligands stabilize the protein structure, similar to the inhibitors RM5 and MCC950. Regions with lower RMSF, such as Arg43, Val94 to Ile101, Arg123 to His131, Thr216 to Val220, Ile278 to Val281, Ile441 to Arg445, Leu495 to Tyr499, Thr306, and Met528, indicate lower fluctuations and, therefore, greater stability. This hypothesis is corroborated by the energy contribution analysis, which highlights these residues as essential for the stabilization of natural compounds in the NLRP3 allosteric site.

Lower Rg values for MCC950, Catechin, Epicatechin, and RM5 indicate a more compact and stable conformation of the protein. The compaction suggests that the binding of these ligands induces an inactive conformation of the protein, corroborating the findings of Casali et al. [46]. It is worth noting that Catechin and Epicatechin stood out with the lowest binding energies among the natural compounds from açaí.

The correlation of these data provides a deeper understanding of the stability and interactions of ligands with the NLRP3 protein. These analyses offer valuable insights into how different ligands influence the structure and function of the protein, potentially leading to the development of new effective inhibitors based on compounds derived from açaí. This study complements previous research and highlights the importance of natural compounds in inhibitor discovery, contributing to the advancement of safer and more effective therapies for inflammatory diseases.

### 2.5. PROTOX 3.0

Table 3 provides a detailed overview of the toxicity of the studied natural molecules, including parameters such as LD50, hepatotoxicity, carcinogenicity, mutagenicity, and cytotoxicity. These parameters are crucial for assessing the safety and feasibility of the compounds as potential NLRP3 inflammasome inhibitors.

LD50 represents the dose required to cause the death of 50% of a test population. This value is a fundamental measure of acute toxicity, and in the context of this study, LD50 values were classified into different toxicity classes according to the following categorization: Class 1: LD50 ≤ 5 mg/kg; Class 2: 5 < LD50 ≤ 50 mg/kg; Class 3: 50 < LD50 ≤ 300 mg/kg; Class 4: 300 < LD50 ≤ 2000 mg/kg; Class 5: 2000 < LD50 ≤ 5000 mg/kg; Class 6: LD50 > 5000 mg/kg.

The hepatotoxicity parameter assesses the potential of a substance to cause liver damage, the organ responsible for detoxification. Carcinogenicity refers to the ability of a substance to cause cancer. The evaluation of this parameter is essential to determine the risks of tumor development due to prolonged exposure. Meanwhile, mutagenicity evaluates the ability of a substance to cause genetic mutations, which can result in cancer or genetic defects. Cytotoxicity measures the toxicity of a substance in cells, indicating the potential to cause cellular damage. The values presented in Table 3 offer a detailed view of the toxicity of the studied natural molecules.

The values in parentheses, such as 0.87, represent the confidence level of the prediction provided by the in silico model. Higher values indicate greater confidence in the predictions. Generally, values above 0.70 are considered reliable, while lower values suggest the need for additional studies for confirmation.

Despite the more favorable binding free energies of the known inhibitors MCC950 and RM5, the high toxicity associated with these compounds is a significant concern. These inhibitors have shown therapeutic potential in treating inflammatory diseases, such as autoimmune and neurodegenerative diseases, due to their ability to modulate the NLRP3 inflammasome. However, significant adverse effects, including hepatotoxicity and other side effects, limit their clinical applicability.

In contrast, natural inhibitors derived from açai, such as apigenin, catechin, and epicatechin, demonstrate substantially lower toxicity, with high LD50 values and inactivity in multiple toxicity parameters (see Table 3). This justifies the search for new NLRP3 inhibitors that combine therapeutic efficacy with a superior safety profile, such as the natural compounds studied in this work.

Additionally, natural compounds are bioavailable, meaning they are easily absorbed and utilized by the body, while known inhibitors are synthetic products. This lower toxicity, combined with sustainability, bioavailability, and potential synergistic effects of natural bioactive compounds, positions these inhibitors as promising alternatives.

Among the natural compounds analyzed, catechin and epicatechin stand out as the most promising. Both exhibit very favorable toxicity profiles, classified in Class 6 with an LD50 of 10,000 mg/kg and relatively favorable binding free energies of −20.1292 kcal/mol and −18.7737 kcal/mol, respectively. Therefore, although the binding affinity of natural products may be lower, their superior safety profile and additional benefits justify their development and use as safer alternative therapies.

This analysis provides a comprehensive view of the evaluated toxicity parameters and highlights the most promising natural molecules based on the reliable predictions of the in silico models used in the study.

## 3. Materials and Methods

### 3.1. Obtaining the 3D Structure of the Receptor

The structure of NLRP3 was retrieved from the Research Collaboratory for Structural Bioinformatics Protein Data Bank database and deposited under code 7ALV. Some discontinuities in the structure were corrected through homology molecular modeling using the SWISS-MODEL server. The generated model was validated using the Ramachandran plot (See Supplementary Materials).

### 3.2. Obtaining the Ligands’ Structure

The crystallographic ligand RM5 (NP3-146) was extracted from the structure of NLRP3. The remaining major constituents of the chemical matrix in the hydroalcoholic extract of açaí were obtained from PubChem, from where the structure of MCC950 was also obtained. The chemical matrix comprises A-MCC950 (CID 9910393); B-Gallic Acid (CID 370); C-Catechin (CID 9064); D-Chlorogenic Acid (CID 1794427); E-Caffeic Acid (CID 689043); F-p-Coumaric Acid (CID 637542); G-Epicatechin (CID 72276); H-Orientin (CID 5281675); I-Luteolin (CID 5280445); and J-Apigenin (CID 5280443).

To accurately represent the electrostatic interactions of the ligands during the molecular dynamics simulations, we performed quantum mechanical (QM) optimization only on the ligands using Gaussian 09. This step was essential to obtain the RESP (Restrained Electrostatic Potential) charges for the ligands. These charges are crucial for ensuring the precision of electrostatic interactions in molecular dynamics models.

The QM optimization was applied exclusively to the ligands and not to the entire protein-ligand-solvent system, as optimizing the entire system would be excessively time-consuming and unnecessary. After the ligands were optimized, the complete system was prepared for molecular dynamics simulations, ensuring that electrostatic interactions were accurately represented without the need for whole-system optimization.

### 3.3. Redocking

Our docking protocol was validated through a redocking process. This involved comparing the RMSD of the docked poses with the experimental poses obtained from crystallographic data. Specifically, the inhibitor RM5 was again docked into the active site of NLRP3, resulting in an RMSD of 0.391 Å. Traditionally, RMSD values below 2.0 Å are considered indicative of a robust docking protocol, depending on the size and flexibility of the ligand. Our result confirms the accuracy and reliability of our docking protocol. The docking simulations were performed using Molegro Virtual Docker (MVD) with the following parameters based on the literature: X = 17.03; Y = 35.46; Z = 125.55; the grid resolution: 0.30 Å; the number of runs: 10; the population size: 50; the maximum iterations: 1500; the energy threshold: 100.0; and the binding site radius (around the active site): 15 Å. These parameters were selected to balance computational efficiency and accuracy, ensuring reliable docking results. The RMSD threshold for validation was set at 2.0 Å, consistent with established practices in molecular docking studies. This rigorous validation process demonstrates that our docking protocol is robust and reliable.

### 3.4. Molecular Docking

Once the docking protocol was validated, the ten compounds from the hydroalcoholic extract of açaí were subjected to virtual screening along with the potent inhibitor MCC950, using the same parameters as the redocking. One conformation was generated for each structure submitted to docking.

### 3.5. Molecular Dynamics Simulations

The protocol used for simulations after the formation of complexes resulting from molecular docking was conducted as follows: The tLEaP module of the AMBER package [81] solvated the system in a rectangular box with periodic boundary conditions, adding water molecules using the TIP3P model [82,83], and adding counter ions to neutralize system charges. The complexes underwent QM optimization at the HF/6-31G* level using the Gaussian09 program [84]. Then, at the same QM level, partial atomic charges for all docked compounds were obtained using the RESP method [85]. The AMBER force field (GAFF) and AMBER ff14SB [86] were used to describe parameters for ligands and enzymes, respectively. The H++ server [87] was used to calculate the protonation states of amino acid residues at neutral pH.

In preparing the protein-ligand-solvent system for molecular dynamics simulations, we ensured that the system was neutralized to maintain electrostatic stability and avoid artifacts. Additionally, to reflect physiological conditions, we added ions to reach a 0.15 M physiological concentration. This step ensures that the ionic strength of our system accurately mimics physiological conditions, providing more biologically relevant results.

Subsequently, the formed systems underwent 4 stages of energy minimization using the NCYC method. This method blended the Steepest Descent and Conjugate Gradient methods [88] to energetically optimize atomic coordinates. Half of the steps were calculated with Steepest Descent, and then the algorithm switched to Conjugate Gradients and continued with this method until the end of the steps. The first stage was solvent relaxation, the second was hydrogen relaxation of the protein, the third was simultaneous relaxation of protein and solvent hydrogens, and finally, a general system minimization.

In the production phase, 100 ns simulations were performed for each system under constant temperature and pressure (NPT). The trajectories obtained in this phase were analyzed and used as a basis for binding free energy calculations.

### 3.6. Generalized Born and Surface Area Continuum Solvation (MM/GBSA)

To elucidate the binding affinity of each bioactive molecule and the crystallographic inhibitor with the NLRP3 protein, we conducted binding free energy calculations employing the MM/GBSA method [89] incorporated in the AmberTools23 package [90]. The mathematical framework for this approach has been previously detailed in published works [63]. The determination of binding free energy and its decomposition was performed based on the last 10 ns of MD simulation trajectories.

### 3.7. In Silico Toxicology

The evaluation of a compound’s toxicity is a crucial step in the development of new drugs. In silico toxicity studies offer a faster and more economical method compared to in vivo toxicity tests conducted on animals, significantly contributing to the reduction of the number of animals used in experiments [91,92].

ProTox 3.0 is an online tool designed to predict acute oral toxicity in rats (https://tox.charite.de/protox3/index.php?site=compound_input, accessed on 20 May 2024). Its toxicity models use two-dimensional similarity analyses between the queried compounds and those in the library, whose LD50s (median lethal dose estimate where 50% of the test subjects die when exposed orally) are known. Additionally, the tool classifies compounds into different toxicity classes based on this parameter [93,94].

Furthermore, ProTox 3.0 predicts various toxicity parameters such as hepatotoxicity, cytotoxicity, carcinogenicity, and mutagenicity. These predictions are based on in vivo study results and in vitro assays contained in the tool’s database [93,94].

## 4. Conclusions

This research investigated the anti-inflammatory potential of natural compounds from açaí (*Euterpe oleracea* Mart.) as inhibitors of the NLRP3 inflammasome, using advanced computational approaches, including molecular docking, molecular dynamics simulations, and binding free energy calculations. The redocking analysis with the RM5 ligand generated an RMSD of 0.391 Å, confirming the accuracy of the docking protocols used and the ability of the Molegro Virtual Docker 5.5 software to reproduce satisfactory experimental results. The natural compounds from açaí showed a significant affinity for the allosteric site of NLRP3. Compounds such as catechin, apigenin, epicatechin, and luteolin stood out for their relatively favorable binding energies and interactions with key residues, similar to the known inhibitors RM5 and MCC950. The docking interaction analysis revealed that the natural compounds exploit both common and unique residues, suggesting potential for the discovery of new inhibitors with unique properties.

Molecular dynamics simulations showed that the presence of natural compounds stabilizes the NLRP3 structure, as indicated by the RMSD, SASA, and Radius of Gyration values. Catechin and epicatechin exhibited behaviors similar to the inhibitors RM5 and MCC950, inducing more compact and stable conformations. Luteolin, despite having a higher RMSD, converged to values lower than the APO form at the end of the simulation. Additionally, luteolin displayed a distinct behavior by increasing the protein’s radius of gyration, suggesting a more open conformation. This unique behavior of luteolin will be investigated in future studies to elucidate its activation mechanisms and potential as an allosteric modulator of NLRP3.

Predictive toxicity analysis using PROTOX 3.0 highlighted that the natural compounds from açaí have significantly lower toxicity compared to synthetic inhibitors. Catechin and epicatechin demonstrated favorable safety profiles, classified in toxicity class 6 with high LD50 values. The results obtained support the hypothesis that natural compounds from açaí have promising therapeutic potential as inhibitors of the NLRP3 inflammasome. The lower toxicity associated with these compounds, coupled with their ability to conformationally stabilize the target protein, positions them as viable candidates for the development of new anti-inflammatory therapies.

This research paves the way for future studies that should include in vitro and in vivo evaluations of the natural compounds to confirm the anti-inflammatory efficacy and safety observed in computational predictions. Additional investigations into the modulation of NLRP3 inflammasome activity by natural compounds, exploring their molecular interactions and detailed mechanisms of action, and the development of derivatives and analogs of the studied compounds to optimize their pharmacological properties and maximize therapeutic efficacy.

In summary, this study not only contributes to the understanding of the pharmacological potential of açaí compounds but also highlights the importance of computational approaches in accelerating and enhancing the drug discovery process from natural resources.

## Figures and Tables

**Table 1:** RMSD, SASA, and Radius of Gyration for NLRP3 Complexes. This table presents the Root Mean Square Deviation (RMSD), Solvent Accessible Surface Area (SASA), and Radius of Gyration (Rg) values for NLRP3 in its apo form and when bound to various ligands. RMSD values indicate structural stability, SASA values reflect solvent exposure, and Rg values describe the compactness of the protein. Lower RMSD and SASA values, along with decreased Rg, suggest that the binding of ligands like Catechin and Epicatechin stabilizes and compacts the NLRP3 structure, reducing its solvent exposure.

	Root Mean Square Deviation (RMSD)		Solvent Accessible Surface Area (SASA)		Radius of Gyration (Rg)	
System	Average (Å)	Standard Deviation (Å)	Average (Å^2^)	Standard Deviation (Å^2^)	Average (Å)	Standard Deviation (Å)
APO	4.41	0.71	27,634.13	497.17	24.35	0.21
Apigenin	3.75	0.47	26,069.83	501.02	24.21	0.12
Caffeic Acid	3.41	0.40	26,224.30	442.52	24.26	0.14
Catechin	3.12	0.37	25,698.55	502.00	23.96	0.08
Chlorogenic Acid	3.88	0.36	26,859.50	594.17	24.39	0.13
Epicatechin	3.59	0.67	26,324.49	558.62	23.98	0.10
Gallic Acid	3.30	0.33	26,463.30	663.70	24.15	0.08
Luteolin	4.38	0.58	26,876.41	640.25	24.73	0.25
MCC950	3.48	0.27	26,370.41	434.62	23.94	0.12
Cyanidin-3-O-Glucoside	3.52	0.70	26,695.52	444.48	24.19	0.10
Orientine	3.80	0.64	26,246.06	495.97	24.38	0.17
RM5	3.04	0.38	26,220.10	413.94	23.97	0.08
Coumaric Acid	3.12	0.35	26,778.23	525.22	24.04	0.15

**Table 2:** MM/GBSA Binding Free Energy Calculations. This table presents the binding free energy (ΔG_bind_) values for the natural compounds from açaí and known inhibitors RM5 and MCC950 when bound to NLRP3. Lower values indicate higher binding affinity. Catechin and Epicatechin exhibit favorable binding energies, suggesting their potential as effective NLRP3 inhibitors.

Molecule	∆E_vdW_	∆E_ele_	∆G_GB_	∆G_nonpol_	∆G_MM/GBSA_
Apigenin	−28.6649 ± 0.0667	−14.0055 ± 0.1175	27.1086 ± 0.0710	28.9187 ± 0.0036	−19.8057 ± 0.0532
Caffeic Acid	−19.3872 ± 0.0400	−22.8664 ± 0.0870	36.3582 ± 0.0656	−17.9832 ± 0.0114	−9.6256 ± 0.0468
Catechin	−29.6376 ± 0.0612	−45.9005 ± 0.1412	60.1916 ± 0.0960	−25.6636 ± 0.0159	−20.1292 ± 0.0668
Chlorogenic Acid	−30.0521 ± 0.0765	−36.6544 ± 0.4053	58.4640 ± 0.2546	−28.2689 ± 0.0221	−13.9546 ± 0.1377
Cyanidin 3-O-glucoside	−25.6313 ± 0.0952	−59.9164 ± 0.5827	80.2020 ± 0.4770	−26.3781 ± 0.0471	−10.4675 ± 0.1005
Epicatechin	−26.0321 ± 0.0754	−54.0411 ± 0.1159	65.9256± 0.0739	−25.5612 ± 0.0163	−18.7737 ± 0.0654
Gallic Acid	−14.4010 ± 0.0637	−52.2527 ± 0.1053	55.2841 ± 0.0563	−16.0951 ± 0.0119	−14.6333 ± 0.0559
Luteolin	−26.3884 ± 0.0708	−44.0228 ± 0.1869	56.0139 ± 0.1392	−23.2228 ± 0.0173	−18.7331 ± 0.0612
MCC950	−50.9995 ± 0.0653	−43.5026 ± 0.1258	59.0414 ± 0.0685	−38.4724 ± 0.0193	−42.4209 ± 0.0908
Orientin	−33.6420 ± 0.0890	−36.4089 ± 0.3689	64.2372 ± 0.2527	−30.3262 ± 0.0420	−11.8606 ± 0.1180
RM5	−50.6779 ± 0.0688	−42.1638 ± 0.1174	53.4280 ± 0.0842	−37.9721 ± 0.0222	−46.5965 ± 0.0743
*p*-coumaric Acid	−19.2039 ± 0.0514	−17.1580 ± 0.1335	26.8670 ± 0.0992	−17.8622 ± 0.0131	−13.1630 ± 0.0521

**Table 3:** Predicted Toxicity of Compounds Using PROTOX 3.0. This table details the predicted toxicity parameters for various compounds, including their LD50 values and activity in hepatotoxicity, carcinogenicity, mutagenicity, and cytotoxicity. Compounds like Catechin and Epicatechin show lower toxicity, making them promising candidates for safe therapeutic use.

Molecule	Predicted LD50	Predicted Toxicity Class	Hepatotoxicity	Carcinogenicity	Mutagenicity	Cytotoxicity
Apigenin	2500 mg/kg	5	Inactive (0.68)	Inactive (0.62)	Inactive (0.57)	Inactive (0.87)
Caffeic Acid	2980 mg/kg	5	Inactive (0.57)	Active (0.78)	Inactive (0.98)	Inactive (0.86)
Catechin	10,000 mg/kg	6	Inactive (0.72)	Inactive (0.51)	Inactive (0.55)	Inactive (0.84)
Chlorogenic Acid	5000 mg/kg	5	Inactive (0.72)	Inactive (0.68)	Inactive (0.93)	Inactive (0.80)
Cyanidin 3-O-glucoside	5000 mg/kg	5	Inactive (0.76)	Inactive (0.86)	Inactive (0.72)	Inactive (0.59)
Epicatechin	10,000 mg/kg	6	Inactive (0.72)	Inactive (0.51)	Inactive (0.55)	Inactive (0.84)
Gallic Acid	2000 mg/kg	4	Inactive (0.61)	Active (0.56)	Inactive (0.94)	Inactive (0.91)
Luteolin	3919 mg/kg	5	Inactive (0.69)	Active (0.68)	Active (0.51)	Inactive (0.99)
MCC950	1600 mg/kg	4	Active (0.55)	Inactive (0.69)	Inactive (0.77)	Inactive (0.71)
Orientin	1213 mg/kg	4	Inactive (0.81)	Inactive (0.72)	Active (0.52)	Inactive (0.87)
RM5	1600 mg/kg	4	Active (0.55)	Inactive (0.67)	Inactive (0.82)	Inactive (0.79)
*p*-coumaric Acid	2850 mg/kg	5	Inactive (0.51)	Active (0.50)	Inactive (0.93)	Inactive (0.81)

## Data Availability

Data are contained within the article and Supplementary Materials.

## Supplementary Materials

The following supporting information can be downloaded at: https://www.mdpi.com/article/10.3390/ijms25158112/s1.
