# Supplementary material for: High-Throughput Molecular Modeling and Evaluation of the Anti-Inflammatory Potential of Açaí Constituents against NLRP3 Inflammasome
Source: Int J Mol Sci. 2024 Jul 25;25(15):8112. doi: 10.3390/ijms25158112 (PMC11311378; doi:10.3390/ijms25158112)
Supplement: Supplementary file 1 [file ijms-25-08112-s001.zip › ijms-3075152-supplementary.pdf]

MolProbity Ramachandran analysis

model_01.pdb, model 1

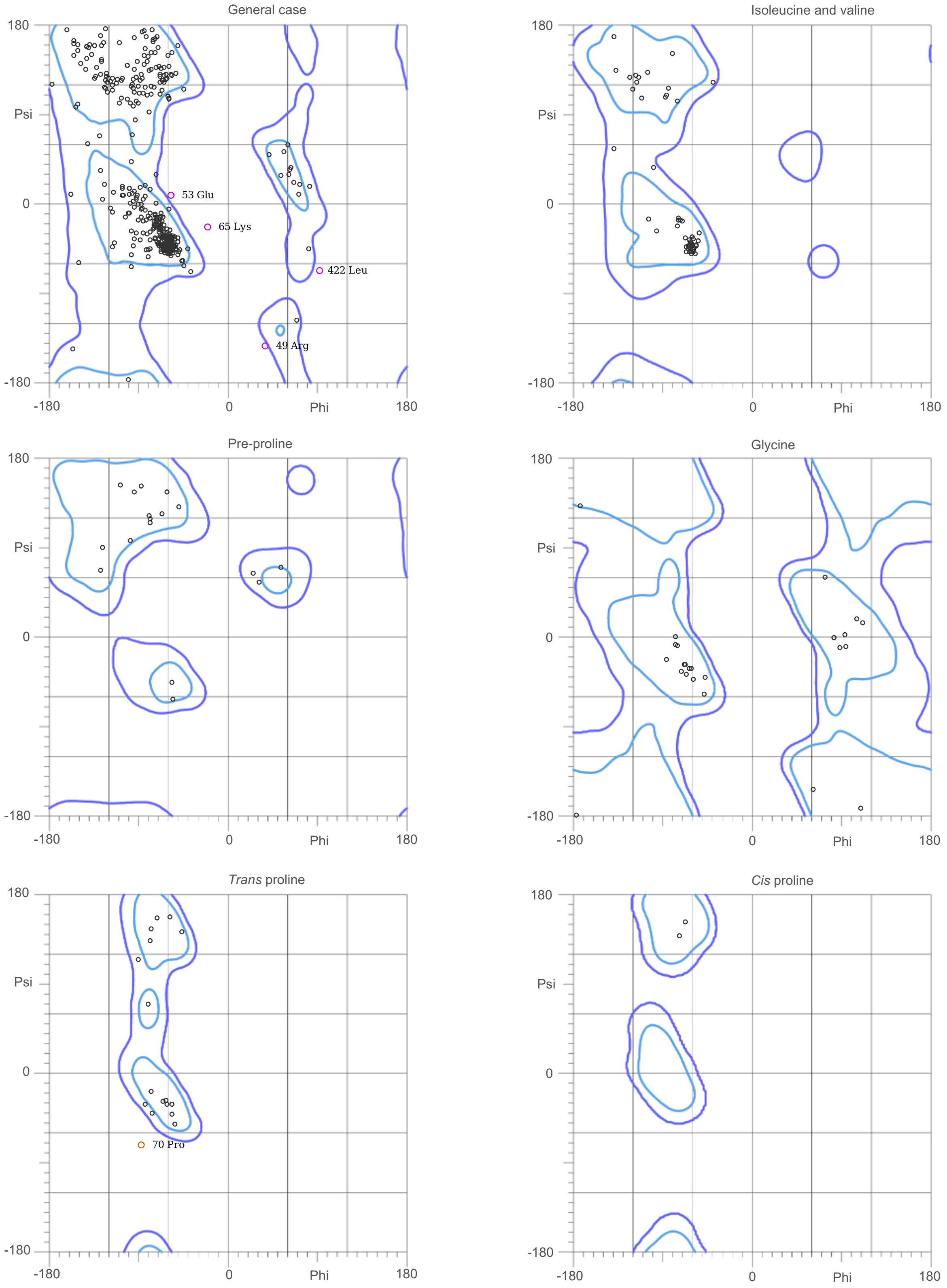

93.2% (504/541) of all residues were in favored (98%) regions.
99.1% (536/541) of all residues were in allowed (>99.8%) regions.

There were 5 outliers (phi, psi):
- 49 Arg (37.2, -143.2)
- 53 Glu (-58.2, 9.4)
- 65 Lys (-21.6, -23.2)
- 70 Pro (-88.2, -72.0)
- 422 Leu (92.1, -67.8)
